# Sensory interference shapes habitat suitability for an acoustically specialized predator

**DOI:** 10.1038/s41598-025-30437-z

**Published:** 2025-12-04

**Authors:** Aleena R. Habib, Julianna M. A. Jenkins, Natalie M. Rugg, Guillermo Alvarez-Nuñez, Damon B. Lesmeister

**Affiliations:** 1 Oak Ridge Institute for Science and Education, Oak Ridge, TN USA; 2 Pacific Northwest Research Station, USDA Forest Service, Corvallis, OR USA; 3 Department of Fisheries, Wildlife, and Conservation Sciences, Oregon State University, Corvallis, OR USA

**Keywords:** Anthropogenic noise, Sensory ecology, Occupancy modeling, Passive acoustic monitoring, Northern saw-whet owl, Ecology, Ecology, Zoology

## Abstract

Ambient acoustic conditions shape how animals perceive and interact with their environments, yet their role in structuring space use remains underexplored. Noise can mask biologically informative sounds which can impact foraging success, physiological fitness, and displace animals from viable habitat. Here, we test how landscape variables and soundscape characteristics across a mixed-use forest landscape affect spatial distribution of a nocturnal predator with specialized hearing. We used passive acoustic recordings to assess the effects of noise levels within biologically relevant frequency ranges on landscape use of the northern saw-whet owl (*Aegolius acadicus*) across 276 sites in Oregon, USA. Owl landscape use declined with increasing noise levels in the 1.60–7.10 kHz band, corresponding with species peak auditory sensitivity. In contrast, general low-frequency sound (0.25–1.00 kHz) was a poor predictor of landscape use but negatively affected acoustic detection probability. These results provide evidence that sensory masking from ambient soundscapes can constrain the realized acoustic niche and drive avoidance of otherwise suitable habitat. Our findings highlight the importance of considering full-spectrum acoustic environments in spatial ecology and suggest that species distributions are shaped not only by physical habitat but also by the perceptual accessibility of ecological information.

**Supplementary Information:**

The online version contains supplementary material available at 10.1038/s41598-025-30437-z.

## Introduction

Acoustic conditions are a fundamental, but often overlooked, dimension of animal habitats. The ambient soundscape, composed of geophony (e.g., wind, water), biophony (e.g., insects, birds, amphibians), and anthrophony (e.g., traffic, machinery), influences how animals detect prey, avoid predators, communicate, and navigate^1–3^. These soundscape components are often spatially and temporally dynamic and may vary considerably across structurally similar habitats. While anthropogenic noise has received the most attention as a disruptive force, natural and biological acoustic signals can also generate masking effects, particularly in species with narrow or highly tuned hearing ranges^4–6^. Despite this complexity, ecological models rarely incorporate the sensory accessibility of habitat and instead assume that structurally suitable areas are functionally usable.

However, this assumption fails for specialized species that rely on sound to interact with their environment. In such cases, landscape use may be constrained not by resource availability or physical barriers, but by the acoustic properties of the environment relative to the species’ sensory system^7^. The field of sensory ecology emphasizes that information is central to survival and fitness, and that environmental conditions that interfere with perception can impose ecological costs as significant as habitat loss or fragmentation^8,9^. For auditory dependent species, masking from background noise—regardless of its source—can reduce signal detection, compromise foraging or mating success, and alter patterns of space use and persistence^10,11^.

Studies have documented vocal compensation behaviors—such as increasing amplitude or shifting pitch—in birds, frogs, and marine mammals exposed to noise^12,13^. However, such compensation may be unavailable to species that use passive auditory cues, such as most predators. For many organisms, anthropogenic noise directly competes with ecologically important sounds, like prey cues or conspecific calls, without an opportunity for adjustment. When noise overlaps with the frequency range or detection thresholds of these species, it can reduce foraging success, and exclude individuals or populations from noisy areas^14,15^.

From a biological perspective, the effects of noise depend not only on its intensity, but on its spectral characteristics and temporal structure, relative to the perceptual system of the organism. For instance, low-frequency traffic noise may have negligible impact on the communication of species that vocalize or hear in high frequencies but could severely impair those with overlapping sensitivity. This highlights the need to treat noise as a general disturbance and align its measurement with the sensory ecology of the species being studied^3,16^.

We argue the need to evaluate resource use within the organism’s realized acoustic niche, the portion of the soundscape where ambient sound conditions allow an organism to perceive critical information about its environment without interference from other species. This niche is defined not only by signal transmission, but also signal reception, shaped by both background noise and species-specific auditory sensitivity^17^. Just as thermal or hydric tolerances define physiological niche boundaries, auditory pressures in the environment may determine where a species can function ecologically. The realized acoustic niche thus complements existing niche frameworks and calls for the integration of sensory constraints into models of habitat suitability.

**Fig. 1:**
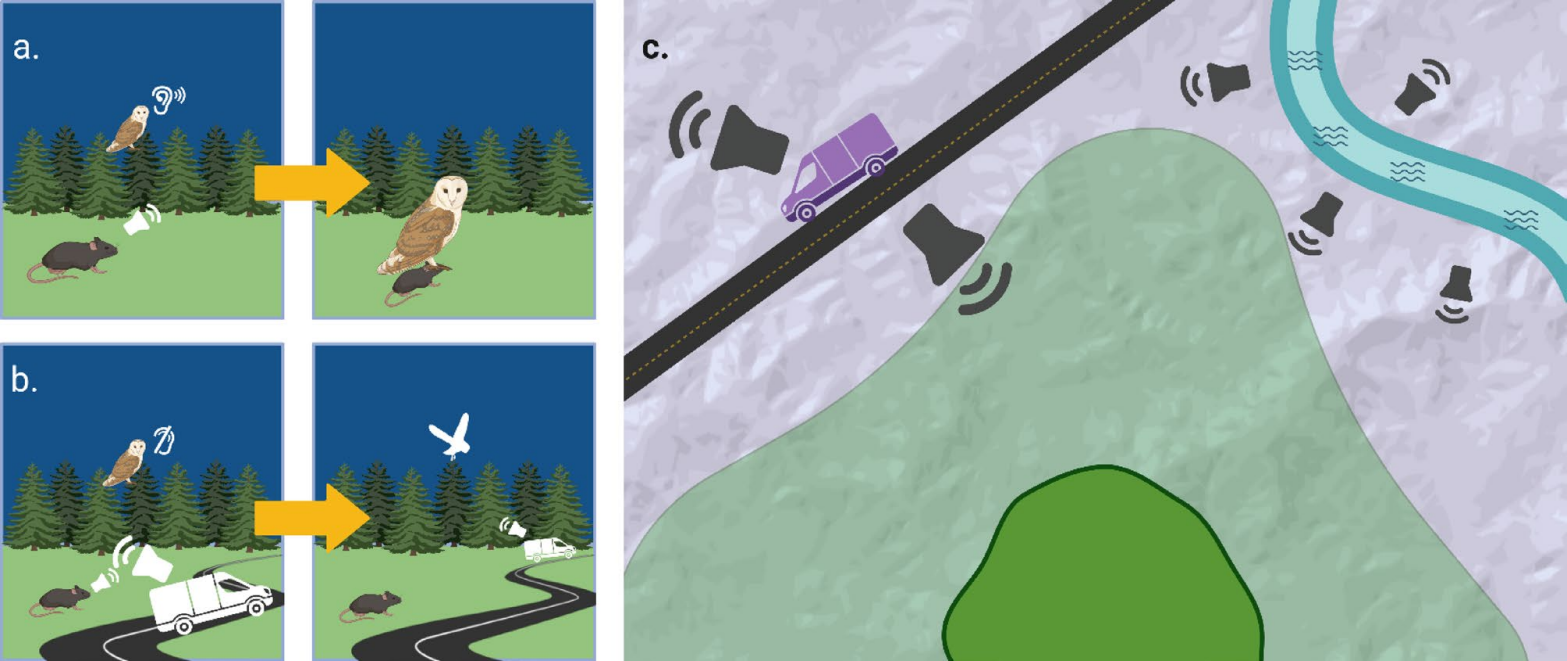
Ideal acoustic foraging conditions free of sensory interference where signals from prey can be detected by the owl, leading to successful prey capture (a). Sensory interference from anthropogenic noise interferes with prey detection and causes the owl to leave the area (b). Sound driven habitat avoidance of the owl caused by sensory interference (c). Owls abandon otherwise suitable habitats due to unsuitable acoustic conditions for foraging and communication. Light green represents suitable habitat while the dark green represents the realized habitat used due to sound driven habitat avoidance (c). Visuals created in Biorender^45^.

The process whereby species are excluded from parts of their structurally suitable habitat due to masking or interference from noise can be considered sound driven habitat avoidance. In this view, habitat loss or unsuitability is not solely a matter of vegetation change, fragmentation, or competitive interactions—it can occur when sensory interference prevents organisms from effectively using space. We describe sensory interference, a driver of habitat avoidance, as an inability to receive vital sensory information due to interference from overlapping acoustic signals (Fig. 1). This is particularly important for species with narrow auditory bandwidths or frequency sensitivity, who may be especially vulnerable to acoustic displacement. The potential for acoustics as a critical component of habitat is widespread, yet empirical demonstrations of the impact of sounds on landscape use remain rare—largely because studies have lacked access to both fine-scale noise data and species-specific hearing profiles.

The northern saw-whet owl (*Aegolius acadicus;* hereafter saw-whet owl) offers a compelling model system for testing this hypothesis. This small, nocturnal forest owl is acoustically sensitive and broadly distributed across North America. The saw-whet owl has a vocal repertoire containing eleven vocalizations. These vocalizations range from 1 to 2 kHz except for the female contact call which is around 8.5 kHz. The call analyzed in this study is the male territorial “toot” call. This call is a series of whistled notes which occur at ~ 1,100 Hz with a call rate of two notes per second. Male saw-whet owls vocalize with this call during breeding seasons to attract mates and establish territories. Female saw-whet owls make a version of this call during courtship, but it varies greatly from the male call in speed and pitch. The “toot” vocalization is often made in breeding territories from forest edges where acoustic detection by conspecifics is increased, and in dense forests, both of which are primary foraging habitats for this owl^18,19^. The saw-whet relies on passive acoustic detection to locate small mammal prey ^20,21^.This owl exhibits extreme auditory specialization, including pronounced ear asymmetry which allows for exceptional sound localization, and hearing sensitivity concentrated between 1.6 and 7.1 kHz, with a peak near 4 kHz ^20,22,23^. This auditory sensitivity is highly tuned to low-amplitude vocalizations made by common prey of the saw-whet owl, which range from 1.5 to 10.2 kHz^20^. These traits support exceptional foraging precision but also make the species vulnerable to mid-frequency noise interference, particularly from railways, machinery, and other sources common in human-modified forests^9,21^.

Passive acoustic monitoring (PAM) is a burgeoning method for monitoring nocturnal and elusive soniferous species with the benefit of being a non-invasive tool to monitor wildlife populations that simultaneously gathers useful metrics for soundscape research. Here, we use > 100,000 h of PAM recordings across 276 forested sites in a low-to-mid elevation industrial forest with a mixed coniferous landscape, to evaluate whether ambient noise within biologically relevant frequency bands predicts variation in landscape use by saw-whet owls. Using a convolutional neural network^24^ to detect owl calls and site-level noise metrics derived from third-octave band analysis, we applied single-season occupancy models to estimate landscape use and acoustic detection probability. We specifically tested whether noise levels in the owl’s sensitive frequency band (1.60–7.10 kHz) were more predictive of landscape use than low-frequency noise (0.25–1.00 kHz), which captures anthropogenic noise and low frequency geophony, anthrophony, and biophony while accounting for vegetation structure, terrain, and detectability. Decreased distance to transportation infrastructure increases habitat degradation, physiological stress, increased risk of collision, and masking of important cues and signals from prey and conspecifics by traffic noise^11,25^. We hypothesized that saw-whet owl landscape use would decrease with proximity to transportation infrastructure and higher levels of noise in the most sensitive hearing frequencies of the saw-whet owl.

## Results

**Fig. 2:**
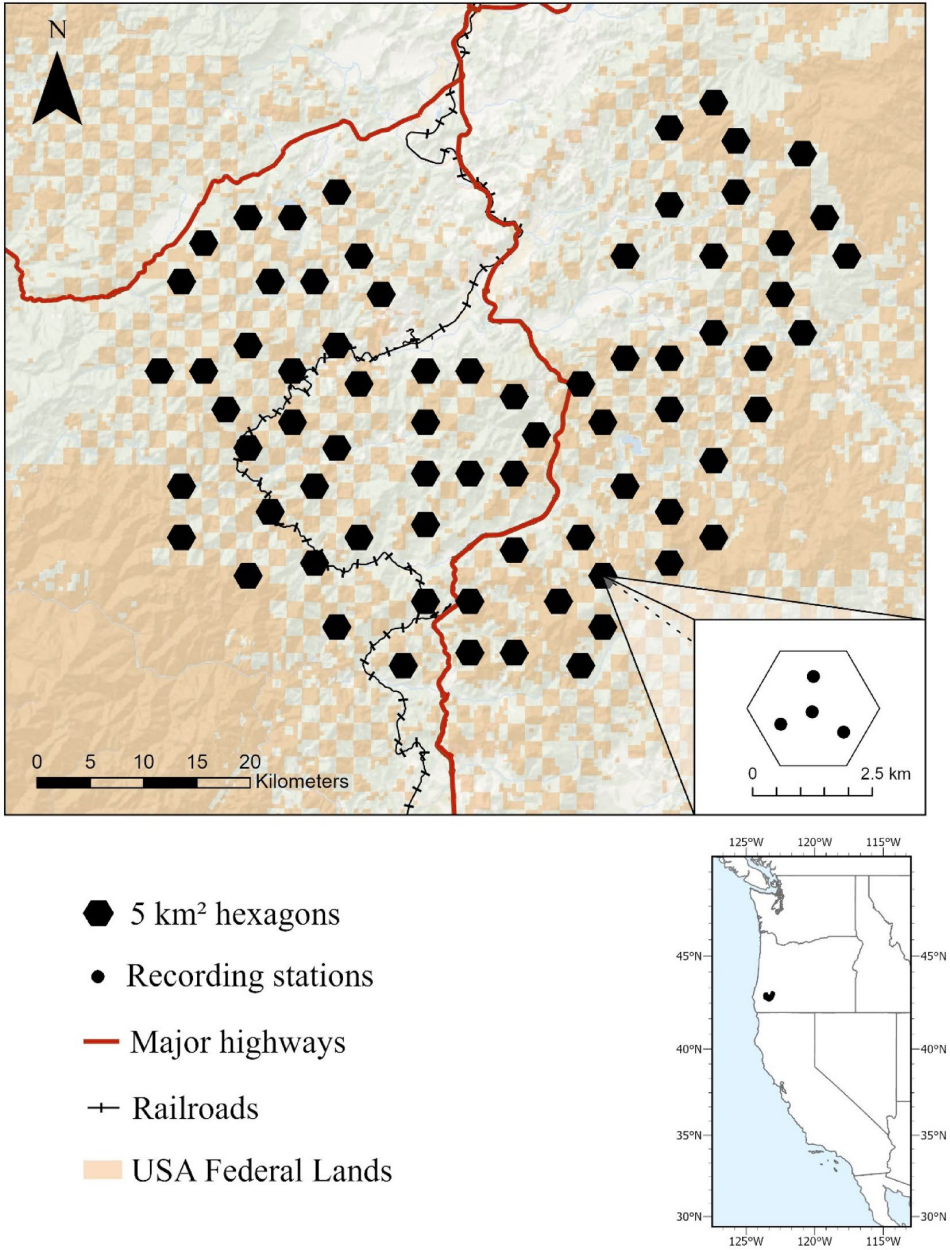
Passive acoustic sampling of 276 recording sites within 69 5-km^2^ hexagonal units in Oregon, USA in 2021. Each randomly selected hexagonal unit was sampled with four autonomous recording stations. Visuals created in ArcGIS Pro^37^.

We collected and processed 105,673.10 h of audio from 276 autonomous recording units (ARUs) from March 2nd to September 21 st, 2021, in a mixed ownership forest landscape in southwestern Oregon, USA (Fig. 2). ARUs recorded for approximately 9 weeks (44.37 ± 0.10 days; mean ± SE). We used a convolutional neural network, PNW-Cnet v4, to identify 12-second audio clips with saw-whet owl territorial ‘toot’ vocalizations^24^. We manually validated saw-whet owl vocalizations at the site level and confirmed presence at 59% (*n* = 163) of sites at sites with confirmed presence, we adjusted predicted counts of nighttime detections by an area-specific classifier precision of 0.86. We used a threshold of 2, 12-second audio clips with apparent detections to assign daily presence in detection histories to limit false positives. Within those sites, we found 113,584 apparent detections of saw-whet owl territorial calls after adjusting for classifier precision. Occupied stations detected owls for 9.07 ± 0.73 days (mean ± SE) with 1–857 apparent acoustic detections per day.

The final candidate model set included a diversity of covariate structures for acoustic detection probability (*p*) and landscape use (ψ) parameters (Table S3). The most-supported model [*p*(LOWFREQNOISE + PRECIP + DATE), ψ(BRDLF_400_ + ELEV + STRMDIST + RAILROAD + OWLFREQ + SMCON_200_)] held 61% of the model weight and outperformed the second-ranked model by 2.34 AICc (Table 1). The six top-ranked models (95% of the cumulative model weight) differed only in ψ sub-model structure (Table 1). A Pearson chi-squared goodness-of-fit test with 1000 bootstraps indicated adequate fit (p-value of 0.46) and no evidence of overdispersion ($\:\widehat{c}$ =1.01)^26^ for our top-ranked model.

**Table 1:** Most-supported single species occupancy models (≥ 0.95 of cumulative model weight) for Northern saw-whet Owl landscape use (ψ) and detection probability (p) ranked by akaike’s information criterion for small sample sizes (ΔAICc), including akaike’s model weight (w), number of parameters (k), and the twice negative log-likelihood (−2LogL). See supplemental materials for full candidate model sets (Tables S5-S7). Detection model structure for all top models was *p*(LOWFREQNOISE + PRECIP + DATE).

Model	k	−2LogL	ΔAICc^a^	w
ψ(BRDLF400 + ELEV + STRMDIST + RAILROAD + OWLFREQ + SMCON200)	11	7309.51	0.00	0.61
ψ(BRDLF400 + ELEV + STRMDIST + RAILROAD + SMCON200)	10	7314.02	2.34	0.19
ψ(BRDLF400 + ELEV + STRMDIST + RAILROAD + OWLFREQ)	10	7315.68	4.00	0.08
ψ(BRDLF400 + ELEV + STRMDIST + RAILROAD)	9	7319.26	5.43	0.04
ψ(BRDLF400 + ELEV + STRMDIST + OWLFREQ)	9	7321.08	7.24	0.02
ψ(BRDLF400 + ELEV + RAILROAD + OWLFREQ)	9	7322.24	8.40	0.01

SMCON_200_ = proportion of small conifers within 200 m, BRDLF_400_ = proportion of broadleaf trees within 400 m, RAILROAD = distance to nearest railroad (km), STRMDIST = distance to nearest stream (m), OWLFREQ = mean noise values within the range of 1.6–7.6.l kHz (dBFS), ELEV = elevation (m), PRECIP = levels of daily precipitation (mm), DATE = Julian date, LOWFREQNOISE = mean nightly noise values calculated from 250–1000 Hz (dBFS).

^a^The AICc of the top ranked model was 7332.512.

Daily acoustic detection probability of saw-whet owl vocalizations was negatively associated with precipitation, low-frequency noise, and date (Table 2). One standard deviation (4.72 dBFS) increase in low-frequency noise was associated with a 33% decrease in odds of acoustic detection. While one standard deviation (2.66 mm) increase in daily precipitation was associated with a 10% decrease in the odds of acoustic detection. The odds of detecting a saw-whet owl decreased by 47% when surveying 32 days later in the breeding season (Table 2).

**Table 2:** Covariate coefficients (β) from top single-species occupancy model for the Northern saw-whet Owl landscape use (ψ) and detection likelihood (*p*) ranked by akaike’s information criterion for small sample sizes, including standard error (SE) the lower and upper 95% confidence intervals (LCL; UCL). All variables were scaled to have a mean of 0 and standard deviation of 1 prior to analysis.

Parameter	Variable ^a^	β	SE	LCL	UCL
*p*	Intercept	−1.67	0.04	−1.74	−1.592
*p*	PRECIP	−0.10	0.03	−0.16	−0.03
*p*	DATE	−0.63	0.04	−0.71	−0.56
*p*	LOWFREQNOISE	−0.40	0.04	−0.48	−0.32
ψ	Intercept	0.44	0.14	0.17	0.71
ψ	BRDLF_400_	−0.52	0.17	−0.85	−0.19
ψ	ELEV	−0.42	0.16	−0.73	−0.12
ψ	STRMDIST	0.38	0.18	0.02	0.74
ψ	RAILROAD	−0.36	0.18	−0.70	−0.01
ψ	OWLFREQ	−0.30	0.14	−0.58	−0.02
ψ	SMCON_200_	0.37	0.15	0.07	0.67

^a^ SMCON_200_ = proportion of small conifer within 200 m, BRDLF_400_ = proportion of broadleaf trees within 400 m, RAILROAD = distance to nearest railroad (km), STRMDIST = distance to nearest stream (m), OWLFREQ = mean noise values within the range of 1.6–7.6. l kHz. ELEV = elevation (m), DATE = Julian date, LOWFREQNOISE = mean nightly noise values calculated from 250–1000 Hz (dBFS), PRECIP = amount of daily precipitation (mm).

**Fig. 3:**
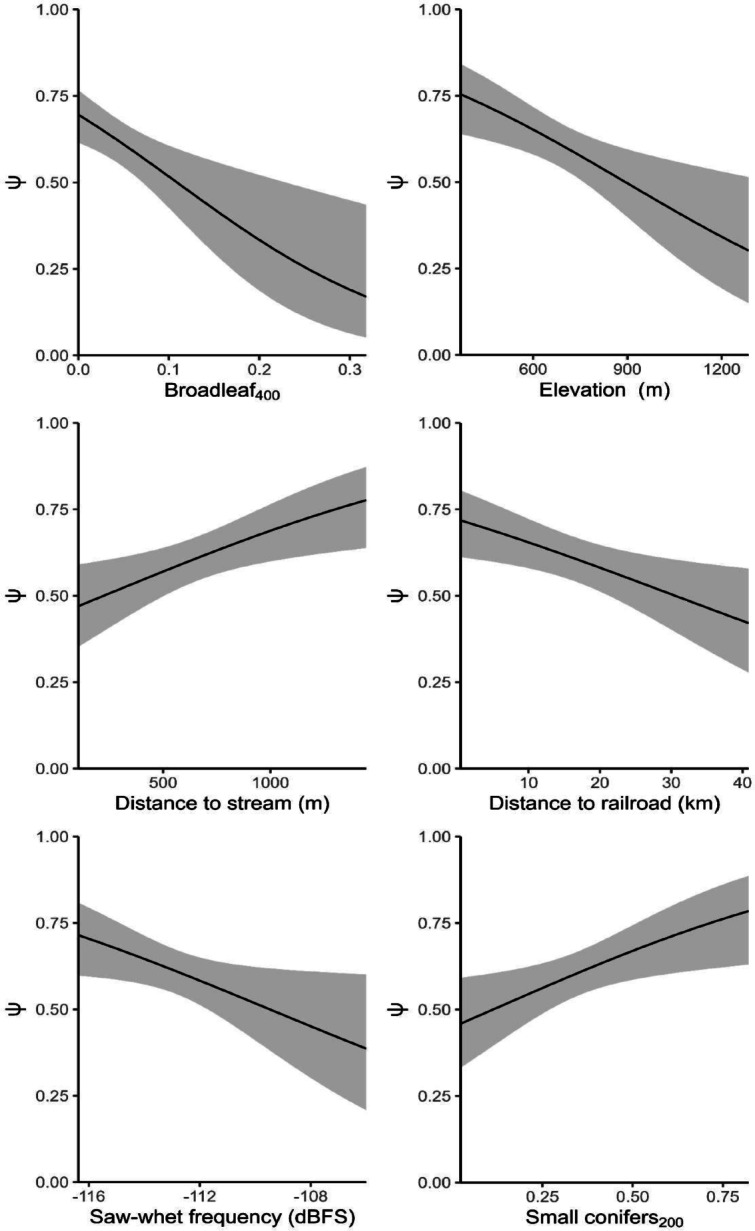
Northern saw-whet owl landscape use probability relative effects of covariates in the most-supported single-season, single-species occupancy model: proportion of broadleaf trees within a 400-meter buffer (BRDLF_400_), elevation of the station in meters (ELEV), distance to nearest stream in meters(STRMDIST), distance to nearest railroad in kilometers (RAILROAD), average nightly frequency levels in decibels relative to full scale between 1.6 and 7.1 kHz (OWLFREQ), and proportion of small conifers within a 200-meter buffer (SMCON_200_). Estimates and 95% confidence limits were generated for each focal covariate while holding other covariates at their mean value.

In addition to strong associations with covariates describing forest and topographical conditions, the top-ranked model of saw-whet owl landscape use (ψ) included a strong negative association with the level of sound within the high sensitivity range of saw-whet owl hearing (OWLFREQ; Table 2; Fig. 3). Each increase of standard deviation in OWLFREQ (2.25 dBFS), decreased the odds of landscape use by 26% (Fig. 3). Low-frequency noise in our study area contained wind and rain, and largely consisted of anthropogenic sounds such as traffic, train noise, mining sounds, and airplane noise. Mid-frequency background (OWLFREQ) noise consisted largely of birds, insects, amphibians, flowing water, intense wind, and intense rain (Figure S1). Average noise levels across all sites in the low frequency noise bands (LOWFREQNOISE) ranged from − 73.3 to −123.5 dBFS, with a mean of −108 ± 4.72 dBFS, while average noise levels across all sites in the most sensitive hearing range of the saw-whet owl ranged from −88.1 to −120.0 dBFS, with a mean of −112.63 ± 2.25 dBFS (OWLFREQ, Table S4).

Saw-whet owl landscape use was also negatively associated with distance to railroad, such that each increase of standard deviation in distance (11.55 km), decreased the odds of landscape use by 30% (Table 2; Fig. 3). Conversely, landscape use was positively associated with distance to stream, where an increase of 370 m (one standard deviation) increased the odds of landscape use by 46% (Table 2; Fig. 3), suggesting that saw-whets may have been attracted to the railroad corridor but avoided areas near streams. Notably there was only one major railway in our study area, which travels through Cow Creek Canyon alongside a large creek (Fig. 2). There was no correlation between distance to railroad (range: 277 − 42,751 m) and any of our seasonal noise covariates. We observed a weak correlation (*r* = −0.17) and slight negative trend in distance to stream (range 83–1779 m) and OWLFREQ noise levels (Table S5, Figure S2-S7.) Saw-whet owl landscape use was negatively associated with elevation (ELEV); where each standard deviation increase (198.63 m) decreased the odds of landscape use by 34.3% (Table 2; Fig. 3). Landscape use was negatively associated with proportion of broadleaf trees within a 400-m buffer (BRDLF_400_) and positively associated with proportion of small conifers within a 200-m buffer (SMCON_200_; Table 2). Each increase of standard deviation in BRDLF_400_ (0.07 m^2^/ha) decreased the odds of landscape use by 41%, while each increase of standard deviation in SMCON_200_ (0.20 m^2^/ha) increased the odds of landscape use by 45% (Table 2; Fig. 3).

## Discussion

Our findings suggest that variation in ambient noise (including geophony, biophony, and anthrophony) across the acoustic spectrum can influence the spatial ecology of an acoustically specialized predator. Saw-whet owl landscape use declined significantly with increasing sound levels within the 1.60–7.10 kHz frequency bands, aligning with the species’ peak auditory sensitivity^20,22^. This frequency-specific effect was not explained by structural habitat features alone, indicating that perceptual accessibility constrains landscape use.

These results offer empirical support for the concept of the realized acoustic niche, a sensory-defined space within which organisms can successfully detect, process, and respond to ecologically meaningful cues^3,7^. Even in structurally suitable environments, species may avoid habitat when ambient sound conditions interfere with their perceptual abilities. We build on this framework by demonstrating how avoidance of suitable habitat arises when sound levels in species-relevant frequency bands mask key ecological information, reducing the functional extent of otherwise viable habitat^11,15^.

The effect of sound on habitat suitability that we observed was not solely attributable to anthropogenic sources. Although many studies in soundscape ecology emphasize roads, aircraft, or urban noise, our study area featured complex acoustic inputs from natural and biological sources. Insects, amphibians, flowing water, and wind all contributed to the mid-frequency background noise measured in our study. Our results emphasize that ecological effects of masking are determined less by source category and more by spectral overlap and spatial-temporal consistency of the sound^1,6^. This perspective recognizes that animals evolved under geophonic and biophonic acoustic pressures and that anthropogenic sounds may add to, rather than replace, existing background noise. In some cases, interactions among sources may have cumulative or non-additive effects on masking or behavioral response^9^. We used seasonally aggregated covariates for occupancy; therefore, the impacts of acute noise events are not fully captured in this analysis.

Saw-whet owls rely on passive detection of conspecific vocalizations and prey-generated cues, such as rustling in leaf litter. Their specialized auditory system includes asymmetric ears and fine frequency discrimination in the 1.60–7.10 kHz range, with peak sensitivity near 4.00 kHz^20^. These traits allow high foraging precision but increase susceptibility to masking in environments where sound levels are elevated in the same frequency range. In contrast to shifting signal amplitude or frequency to compensate for noise, passive-listening predators have fewer compensatory mechanisms^12,21^. Our finding that mid-frequency noise was a better predictor of landscape use than low-frequency noise underscores that spectral specificity is essential in understanding sensory constraints on space use.

While increased distance to railroads had a negative effect on landscape use, this variable may reflect a combination of acoustic and structural influences. Rail corridors can generate high-amplitude yet intermittent noise, but may also support edge habitats for small mammal populations that provide foraging opportunities^27^. The irregularity of this noise may allow periods of auditory recovery or detection, although it may also create unpredictable sensory environments that increase cognitive or energetic costs^15^. Future research could explore whether predictability, amplitude modulation, and periodicity of sound sources mediate the effects of masking across species and behaviors.

Vegetation structure and topography influenced landscape use in expected directions. Areas with higher proportion of small conifers were more likely to be occupied, consistent with previous studies of saw-whet owl nesting and foraging preferences^28^. Elevation had a negative effect, possibly reflecting either prey availability or changing vegetation profiles. Broadleaf proportion was negatively associated with landscape use, which may be due to differences in sound attenuation or prey density in those habitats. Distance to streams had a positive effect, associated with an increase in landscape use further from streams, possibly reflecting different vegetation profiles near riparian areas.

Methodologically, our study demonstrates the value of integrating passive acoustic monitoring with machine learning and species-specific sensory ecology. Automated classifiers such as PNW-Cnet v4 allow large-scale, repeatable acoustic detection of vocalizations for many species, enabling spatial replication at a level difficult to achieve through traditional field methods^29^. While not completely representative of all landscape use by the saw-whet owl, recordings of ‘toot’ calls capture biologically significant areas for territorial saw-whet owls. Female-specific use areas and nonterritorial movements by ‘floater’ owls may be under-represented. When combined with frequency-targeted acoustic metrics and robust occupancy modeling frameworks, this approach facilitates direct testing of hypotheses about how soundscapes influence species distributions and richness^30,31^. As passive acoustic monitoring tools become more accessible, opportunities to quantify perceptual constraints across taxa and ecosystems will expand.

Decreased habitat suitability driven by sound applies not only to owls but to any species that relies on acoustic cues for ecological decision-making. This includes bats that echolocate in narrow frequency bands, frogs that use tonal mating calls, and marine mammals that depend on underwater sound for navigation and foraging^7,32,33^. In each case, the overlap between ambient noise and auditory sensitivity determines whether sensory information is lost or can be accessed. The generality of this mechanism suggests that sensory-based habitat avoidance may be widespread but underutilized in analyses of spatial ecology.

Our results also point to practical implications for conservation. Land managers and conservation planners often rely on structural habitat maps to identify suitable areas for protection or mitigation^34^. However, if perceptual access is impaired, these maps may overestimate functional habitat. For acoustic specialists, it may be necessary to evaluate soundscape conditions within biologically relevant frequency bands as a criterion for habitat quality^7^. Conservation strategies might then include not only habitat restoration but also noise mitigation, timing of human activities, or establishment of acoustic refugia^10,11^.

Future experimental studies could extend these findings by quantifying the effect of acoustic masking from low-frequency noise and mid-frequency noise to reproductive success, energy expenditure, and prey capture rates on the landscape, and whether this leads to acoustic displacement of individuals. Multi-season models incorporating survival or breeding data could help evaluate whether sound driven habitat avoidance has population-level impacts. Additionally, classification of noise sources and spectral structure at higher resolutions would allow researchers to disentangle the relative contributions of different soundscape components. The incorporation of temporal noise into a dynamic occupancy model could help researchers further understand the influence of temporal noise variation on landscape use. Integrating spatial soundscape models with species-specific detections and behavioral data represents a promising frontier for landscape ecology and conservation science.

We found rare evidence that ambient noise, including both natural and anthropogenic components, can influence a species’ use of structurally suitable areas by interfering with its ability to perceive its environment. By demonstrating that sensory constraints shape the realized acoustic niche, we highlight the need to consider not just where animals can survive, but where they can hear. This perspective broadens our understanding of how species interact with dynamic soundscapes and offers a conceptual and methodological foundation for incorporating sensory ecology into spatial modeling, conservation planning, and biodiversity assessment.

## Methods

### Study area

We collected audio data in spring and summer of 2021, within the saw-whet owl’s breeding season, across a 1,422-km^2^ area in southwestern Oregon (Fig. 2) as part of a broad scale PAM program designed for population monitoring of northern spotted owls (*Strix occidentalis caurina*)^29^. This area contains mixed land ownership in a checkerboard of federally managed (U.S. Bureau of Land Management) and private lands. The area is part of the Klamath Mountain region, with a low-to-mid elevation mixed-conifer landscape used for natural resources, including logging, farming, and mining. The Klamath transportation network contains logging roads, secondary highways, and two major transportation networks: Interstate 5 and the Central Oregon and Pacific railroad line that travels through Cow Creek Canyon.

### Data collection

We used acoustic data collected from 276 monitoring stations within 69 randomly selected 5-km^2^ hexagons (Fig. 2)^29^. Approximately 20% of available hexagons (hexagons with ≥ 25% federal ownership and ≥ 50% forest capable) were randomly selected from a tessellation across the area. Each hexagon was sampled using four ARUs (SM4 Wildlife Acoustics, Maynard, MA, USA). Each ARU was deployed on mid to upper slopes, ≥ 500 m from other ARUs, and ≥ 50 m from roads, trails, and streams to reduce excessive noise. ARUs were deployed on small trees for 6–8 weeks. We programmed ARUs to record for four hours during the crepuscular periods and ten minutes on-the-hour every remaining hour of the night and day^29^. The ARUs have the same sensitivity as human hearing, and their listening radius is impacted by factors limiting sound propagation, such as vegetation, terrain, wind, and rain. ARUs had a signal-to-noise ratio of 80 dB typical at 1 kHz, a sampling rate of 32,000 Hz, and a recording bandwidth of 20 Hz to 48 kHz at decibel levels of −33.5 dB to 122 dB.

### Audio processing and validation

We processed audio data using a convolutional neural network (PNW-Cnet v4) trained to identify various acoustic signals using 12-second spectrograms, including the territorial “toot” song of the saw-whet owl^24,35^. To assess the performance of PNW-Cnet v4 for saw-whet owl signals relative to our study area, we randomly selected 200 audio clips with a PNW-Cnet classification score of ≥ 0.95 for manual validation. We estimated classification precision as the number of manually confirmed saw-whet owl calls divided by the total reviewed.

We used each calendar date (i.e., 24-hr period between 00:00 and 23:59) as survey occasions, up to 45 dates per recording station, omitting daytime audio (08:15 to 19:30) when saw-whet owls are unlikely to vocalize. We generated counts of daily apparent nighttime acoustic detections with a classification score of ≥ 0.95 adjusted by our PNW-Cnet v4 precision and used a threshold of the 0.25 quantile of daily acoustic detections to assign survey presence to limit false positives. We manually confirmed presence/absence of saw-whet owl calls at all recording stations. If an occasion did not meet the threshold of apparent acoustic detections but had ≥ 1 manually confirmed acoustic detection(s), then we assigned a positive detection in that survey occasion. If we confirmed zero acoustic detections at a recording station, all survey occasions at the station were assigned non-detection, regardless of the number of PNW-Cnet v4 apparent acoustic detections.

### Survey covariates

We selected survey covariates which we predicted could affect sound attenuation or saw-whet owl calling behavior (Table S4). We used survey occasion Julian date (DATE) to examine if acoustic detection probability changed over the season. We hypothesized that high levels of nightly low-frequency noise would negatively impact our ability to detect owl calls. We calculated low-frequency noise levels from 250 to 1000 Hz in decibels relative to full scale (dBFS) using Kaleidoscope Pro third-octave sound level analysis^36^ to capture sound levels which may mask saw-whet owl calls (LOWFREQNOISE). We further hypothesized that weather conditions may affect owl calling behavior^18^, thus we evaluated daily precipitation (PRECIP) and average daily temperature (TEMP)^37^.

### Site covariates

We calculated site-specific covariates within 200-, 400- and 600-m buffers around ARU locations to evaluate multi-scale variation in landscape use between stations (Table S4)^38^. There is limited information about the home range size of non-migratory populations of saw-whet owls in the Pacific Northwest, so we based our buffer sizes on the likely listening radii of the recording units. We used ArcGIS Pro^39^ and remotely sensed data to calculate landscape and terrain covariates; ruggedness, elevation, and topography(RUGGED, ELEV, TOPO). We used data from a gradient nearest neighbor dataset^40^. to generate forest structure covariates; snag density, stand height, canopy cover, old growth structure index, broadleaf, and small conifers (SNAG, STNDHGHT, CANCOV, OGSI80, BRDLF, SMCON; Table S4). Using data from the Oregon Department of Transportation, we calculated the distance from each recording station to the nearest railroad (RAILROAD). We also calculated the distance from each station to the closest unpaved road, secondary highway, and major highway (UNPAVEDRD, SCNDHWY, MJRHWY). We used data from the National Hydrography Dataset (Version 2.1) to nearest flowlines and non-ephemeral waters (STRMDIST).

We hypothesized that loud noise within the most sensitive frequencies of the saw-whet owl’s hearing range would lead to decreased landscape use and that as the number of loud nights increased, landscape use would decrease. In addition to summarizing seasonal low-frequency noise levels (LOWFREQNOISE), we calculated noise covariates based on the most sensitive frequencies of the saw-whet owl’s hearing range^20^. We ran third-octave sound level analyses^36^ on frequencies between 1.6 and 7.1 kHz (OWLFREQ) and their frequency of best sensitivity, 4 kHz (BESTHZ). We averaged sound level values across nightly audio files for each station. To further investigate if persistent ‘loud’ nights within the low-frequency noise levels and the saw-whet owl’s sensitive hearing frequencies impacted landscape use^41^, we summarized the proportion of survey occasions where noise levels were above the 25% percentile within both low frequencies and the most sensitive areas of the owls hearing range (OWL_LOUD). We used the scale function in R to standardize covariates.

### Data analysis

We fit single-season, single-species occupancy models in package RPresence^41^ to examine saw-whet owl landscape use (ψ) while incorporating variation in daily nighttime acoustic detection probability (*p*)^42^. We used a secondary candidate-set strategy for model selection^43^ and evaluated model support using Akaike’s Information Criterion corrected for small sample sizes (AICc). We developed sub-model sets independently, holding non-focal parameters constant while testing candidate covariates on the focal parameter, progressing to multivariate models using well supported covariates (Table S1–S2). The final candidate model set included combinations of all parameter sub-models within 10 AICc units of the top-ranking sub-model that did not add uninformative parameters (Table S3)^44^. We did not include correlated variables within sub-models (|r| > 0.6; Table S5–S6). We considered coefficient estimates (β) whose 95% confidence intervals (CI) did not include zero as supported. We used a Pearson chi-squared goodness-of-fit test with 1000 bootstraps to evaluate model fit and test for overdispersion^26^.

## Supplementary Information

Below is the link to the electronic supplementary material.

Supplementary Material 1

## Data Availability

The full dataset necessary to run these analyses are publicly available on Zenodo through the following DOI: 10.5281/zenodo.16944516.
