# Supplementary material for: Sensory interference shapes habitat suitability for an acoustically specialized predator
Source: Sci Rep. 2025 Dec 4;16:820. doi: 10.1038/s41598-025-30437-z (PMC12780084; doi:10.1038/s41598-025-30437-z)
Supplement: Supplementary file 1 — Supplementary Material 1 [file 41598_2025_30437_MOESM1_ESM.pdf]

Sensory interference shapes habitat suitability for an acoustically specialized predator

Aleena R. Habib*[1,2], Julianna M. A. Jenkins[2], Natalie M. Rugg[2,3], Guillermo Alvarez-Nuñez[1,2], Damon B. Lesmeister[2,3]

[1] Oak Ridge Institute for Science and Education, Oak Ridge, Tennessee, USA

[2] Pacific Northwest Research Station, USDA Forest Service, Corvallis, Oregon, USA

[3] Department of Fisheries, Wildlife, and Conservation Sciences, Oregon State University, Corvallis, Oregon, USA

* Corresponding author. Email: aleenarhabib@gmail.com

Supplemental Tables

Table S1. Detection probability (*p*) sub-model set of single species occupancy models for northern saw-whet owls ranked by difference in Akaike's information criterion for small sample sizes (ΔAICc), including Akaike's model weight (w), number of parameters (k), and the twice negative log-likelihood (-2LogL). Occupancy model structure (ψ) held at the intercept (1).

Model	k	-2LogL	ΔAICc[a]	w
p(PRECIP + DATE + LOWFREQNOISE)	5	7355.18	0	0.98
p(LOWFREQNOISE + DATE)	4	7365.49	8.23	0.02
p(PRECIP + DATE)	4	7478	120.74	0
p(DATE)	3	7495.35	136.04	0
p(PRECIP + TEMP + LOWFREQNOISE)	5	7504.51	149.33	0
p(TEMP + LOWFREQNOISE)	4	7524.49	167.23	0
p(PRECIP + TEMP)	4	7577.3	220.04	0
p(TEMP)	3	7602.78	243.46	0
p(LOWFREQNOISE)	3	7661.07	301.75	0
p(PRECIP + LOWFREQNOISE)	4	7660.29	303.03	0
p(PRECIP)	3	7697.79	338.47	0
p(1)	2	7700.81	339.45	0

[a] The AICc of the top ranked model was 7365.407

Table S2. Landscape use (ψ) sub-model set of single species occupancy models for northern saw-whet owls ranked by difference in Akaike's information criterion for small sample sizes (ΔAICc), including Akaike's model weight (w), number of parameters (k), and the twice negative log-likelihood (-2LogL). Detection model structure (*p*) held at top detection model: *p*(PRECIP + DATE + LOWFREQNOISE). Models that ranked below the null model (*p*(1) ψ(1)) are not shown.

Model	k	-2LogL	ΔAICc[a]	w
$\psi(BRDLF_{400}$ + ELEV + STRMDIST + RAILROAD + OWLFREQ + $SMCON_{200})$	11	7309.51	0.00	0.61
$\psi(BRDLF_{400}$ + ELEV + STRMDIST + RAILROAD + $SMCON_{200})$	10	7314.02	2.34	0.19
$\psi(BRDLF_{400}$ + ELEV + STRMDIST + RAILROAD + OWLFREQ)	10	7315.68	4.00	0.08
$\psi(BRDLF_{400}$ + ELEV + STRMDIST + RAILROAD)	9	7319.26	5.43	0.04
$\psi(BRDLF_{400}$ + ELEV + STRMDIST + OWLFREQ)	9	7321.08	7.24	0.02
$\psi(BRDLF_{400}$ + ELEV + RAILROAD + OWLFREQ)	9	7322.24	8.40	0.01
$\psi(BRDLF_{400}$ + $SMCON_{200}$ + RAILROAD + BESTHZ)	9	7322.47	8.63	0.01
$\psi(BRDLF_{400}$ + $SMCON_{200}$ + STRMDIST + RAILROAD)	9	7323.37	9.54	0.01
$\psi(BRDLF_{400}$ + ELEV + LOWFREQNOISE + STRMDIST)	9	7324.22	10.38	0.00
$\psi(SMCON_{200}$ + STRMDIST + RAILROAD + OWLFREQ)	9	7325.34	11.51	0.00
$\psi(BRDLF_{400}$ + ELEV + OWLFREQ)	8	7328.01	12.04	0.00

$\psi(BRDLF_{400}$ + ELEV + RAILROAD)	8	7328.11	12.13	0.00
$\psi(BRDLF_{400}$ + RAILROAD + BESTHZ)	8	7328.38	12.41	0.00
$\psi(BRDLF_{400}$ + OWLFREQ + RAILROAD)	8	7328.56	12.58	0.00
$\psi(SMCON_{200}$ + RAILROAD + OWLFREQ)	8	7328.71	12.74	0.00
$\psi(BRDLF_{400}$ + RAILROAD + STRMDIST)	8	7329.20	13.23	0.00
$\psi(BRDLF_{400}$ + $SMCON_{200}$ + RAILROAD + LOWFREQNOISE)	9	7327.37	13.53	0.00
$\psi(BRDLF_{400}$ + BESTHZ + RAILROAD + LOWFREQNOISE)	9	7327.58	13.75	0.00
$\psi(BRDLF_{400}$ + ELEV + LOWFREQNOISE + OWLFREQ)	9	7327.97	14.13	0.00
$\psi(SMCON_{200}$ + BESTHZ + RAILROAD)	8	7330.11	14.14	0.00
$\psi(SMCON_{200}$ + BESTHZ + RAILROAD + LOWFREQNOISE)	9	7328.01	14.18	0.00
$\psi(BRDLF_{400}$ + ELEV)	7	7334.46	16.37	0.00
$\psi(SMCON_{200}$ + STRMDIST + RAILROAD)	8	7332.38	16.41	0.00
$\psi(BRDLF_{400}$ + RAILROAD)	7	7334.70	16.61	0.00
$\psi(BRDLF_{400}$ + RAILROAD + LOWFREQNOISE)	8	7332.63	16.65	0.00
$\psi(BRDLF_{400}$ + BESTHZ + LOWFREQNOISE + STRMDIST)	9	7331.52	17.68	0.00
$\psi(BRDLF_{400}$ + BESTHZ)	7	7336.12	18.02	0.00
ψ(OWLFREQ + $SMCON_{200}$)	7	7336.85	18.76	0.00
$\psi(BRDLF_{400}$ + OWLFREQ)	7	7336.87	18.77	0.00

ψ($SMCON_{200}$ + BESTHZ)	7	7337.54	19.45	0.00
ψ($SMCON_{200}$ + RAILROAD)	7	7337.64	19.55	0.00
ψ($BRDLF_{400}$ + BESTHZ + LOWFREQNOISE)	8	7335.67	19.70	0.00
ψ($BRDLF_{400}$ + STRMDIST)	7	7338.70	20.61	0.00
ψ(OWLFREQ + $CANCOV_{600}$ + ELEV)	8	7336.86	20.89	0.00
ψ($BRDLF_{400}$ + STRMDIST + LOWFREQNOISE)	8	7336.97	21.00	0.00
ψ(RAILROAD + OWLFREQ + $CANCOV_{600}$)	8	7337.89	21.92	0.00
ψ($BRDLF_{400}$ + $SMCON_{200}$)	7	7340.19	22.10	0.00
ψ(STRMDIST + RAILROAD + OWLFREQ)	8	7338.60	22.63	0.00
ψ($BRDLF_{400}$ + $SMCON_{200}$ + LOWFREQNOISE)	8	7338.63	22.66	0.00
ψ($SMCON_{200}$ + ELEV)	7	7340.83	22.73	0.00
ψ($BRDLF_{400}$ + $TOPO_{200}$)	7	7340.99	22.89	0.00
ψ(OWLFREQ + RAILROAD)	7	7341.02	22.93	0.00
ψ(OWLFREQ + ELEV + LOWFREQNOISE + STRMDIST)	9	7336.86	23.03	0.00
ψ($SMCON_{200}$ + STRMDIST)	7	7341.28	23.18	0.00
ψ(STRMDIST + RAILROAD + BESTHZ)	8	7339.28	23.31	0.00
ψ(RAILROAD + BESTHZ + LOWFREQNOISE)	8	7339.36	23.39	0.00
ψ($BRDLF_{400}$)	6	7344.04	23.84	0.00
ψ(OWLFREQ + ELEV)	7	7341.94	23.84	0.00
ψ(OWLFREQ + RAILROAD + LOWFREQNOISE)	8	7339.98	24.00	0.00
ψ(BESTHZ + RAILROAD)	7	7342.24	24.14	0.00
ψ($BRDLF_{600}$)	6	7344.38	24.18	0.00

$\psi(BRDLF_{400}$ + LOWFREQNOISE)	7	7342.43	24.33	0.00
$\psi(BRDLF_{400} + SNAG_{600})$	7	7342.47	24.38	0.00
$\psi(BRDLF_{400} + RUGGED_{200})$	7	7342.54	24.45	0.00
$\psi(ELEV + CANCOV_{600})$	7	7343.04	24.95	0.00
$\psi(OWLFREQ + TOPO_{200})$	7	7343.05	24.95	0.00
ψ(BESTHZ + ELEV)	7	7343.26	25.17	0.00
$\psi(SMCON_{200}$ + LOWFREQNOISE)	7	7343.30	25.21	0.00
$\psi(BESTHZ + CANCOV_{600})$	7	7343.84	25.75	0.00
$\psi(BRDLF_{400} + CANCOV_{600})$	7	7343.90	25.80	0.00
$\psi(BESTHZ + TOPO_{200})$	7	7343.90	25.81	0.00
ψ(STRMDIST + BESTHZ + LOWFREQNOISE)	8	7341.85	25.88	0.00
ψ(ELEV + STRMDIST)	7	7343.98	25.89	0.00
ψ(RAILROAD + LOWFREQNOISE)	7	7344.06	25.96	0.00
ψ(OWLFREQ + STRMDIST)	7	7344.11	26.02	0.00
$\psi(RAILROAD + CANCOV_{600})$	7	7344.16	26.06	0.00
ψ(BESTHZ + STRMDIST)	7	7344.25	26.15	0.00
$\psi(BESTHZ + RUGGED_{200})$	7	7344.30	26.20	0.00
$\psi(SMCON_{200})$	6	7346.42	26.22	0.00
$\psi(OWLFREQ + CANCOV_{600})$	7	7344.34	26.24	0.00
$\psi(RAILROAD + RUGGED_{200})$	7	7344.39	26.29	0.00
ψ(OWLFREQ)	6	7346.56	26.36	0.00
$\psi(SMCON_{200} + CANCOV_{600})$	7	7344.67	26.58	0.00
$\psi(OWLFREQ + RUGGED_{200})$	7	7344.71	26.62	0.00

ψ(STRMDIST + RAILROAD)	7	7344.90	26.81	0.00
ψ($SMCON_{200}$ + $SNAG_{600}$)	7	7345.01	26.91	0.00
ψ(BESTHZ)	6	7347.21	27.01	0.00
ψ(OWLFREQ + LOWFREQNOISE)	7	7346.05	27.96	0.00
ψ($SMCON_{400}$)	6	7348.58	28.38	0.00
ψ(ELEV + LOWFREQNOISE)	7	7346.61	28.51	0.00
ψ(STRMDIST + $CANCOV_{600}$)	7	7346.66	28.57	0.00
ψ(STRMDIST + LOWFREQNOISE)	7	7346.71	28.61	0.00
ψ(RAILROAD + $TOPO_{200}$)	7	7346.86	28.76	0.00
ψ(RAILROAD)	6	7348.97	28.77	0.00
ψ(OWLFREQ + BESTHZ + LOWFREQNOISE)	8	7345.14	29.17	0.00
ψ(ELEV + $RUGGED_{200}$)	7	7347.67	29.58	0.00
ψ(STRMDIST + $SNAG_{600}$)	7	7347.88	29.79	0.00
ψ(ELEV)	6	7350.26	30.06	0.00
ψ($SMCON_{600}$)	6	7350.42	30.22	0.00
ψ(LOWFREQNOISE + $TOPO_{200}$)	7	7348.38	30.29	0.00
ψ($BRDLF_{200}$)	6	7350.56	30.36	0.00
ψ(ELEV + $SNAG_{600}$)	7	7348.59	30.49	0.00
ψ(STRMDIST + $RUGGED_{200}$)	7	7348.67	30.58	0.00
ψ(LOWFREQNOISE + $CANCOV_{600}$)	7	7348.69	30.60	0.00
ψ($CANCOV_{600}$ + $RUGGED_{200}$)	7	7348.82	30.72	0.00
ψ($CANCOV_{600}$ + $TOPO_{200}$)	7	7348.86	30.77	0.00
ψ(STRMDIST)	6	7351.06	30.86	0.00

$\psi(\text{ELEV} + \text{TOPO}_{200})$	7	7349.01	30.91	0.00
$\psi(\text{LOWFREQNOISE})$	6	7351.21	31.01	0.00
$\psi(\text{LOWFREQNOISE} + \text{SNAG}_{600})$	7	7349.29	31.19	0.00
$\psi(\text{CANCOV}_{600})$	6	7351.52	31.32	0.00
$\psi(\text{LOWFREQNOISE} + \text{RUGGED}_{200})$	7	7349.42	31.33	0.00
$\psi(\text{CANCOV}_{400})$	6	7351.54	31.34	0.00
$\psi(\text{RUGGED}_{200} + \text{TOPO}_{200})$	7	7350.12	32.03	0.00
$\psi(\text{RUGGED}_{200})$	6	7352.33	32.13	0.00
$\psi(\text{RUGGED}_{600})$	6	7352.55	32.35	0.00
$\psi(\text{RUGGED}_{400})$	6	7352.68	32.48	0.00
$\psi(\text{RUGGED}_{200} + \text{SNAG}_{600})$	7	7350.64	32.54	0.00
$\psi(\text{TOPO}_{200})$	6	7352.75	32.55	0.00
$\psi(\text{MJRHWY} + \text{LOWFREQNOISE})$	7	7350.65	32.55	0.00
$\psi(\text{SNAG}_{600})$	6	7352.91	32.71	0.00
$\psi(1)$	5	7355.18	32.90	0.00
$\psi(\text{TOPO}_{200} + \text{SNAG}_{600})$	7	7350.99	32.90	0.00
$\psi(\text{OWL\_LOUD})$	6	7353.23	33.03	0.00
$\psi(\text{CANCOV}_{600} + \text{SNAG}_{600})$	7	7351.13	33.03	0.00
$\psi(\text{UNPAVEDRD})$	6	7353.84	33.64	0.00
$\psi(1)\ p(1)$	2	7700.81	372.34	0.00

[a] The AICc of the top ranked model was 7332.512

Table S3. Final candidate set of single species occupancy models combining detection probability (*p*) and landscape use (ψ) sub-models above ten ΔAIC for northern saw-whet owls ranked by difference in Akaike's information criterion for small sample sizes (ΔAICc), including Akaike's model weight (w), number of parameters (k), and the twice negative log-likelihood (-2LogL).

Model	k	-2LogL	ΔAICc[a]	w
ψ($BRDLF_{400}$ + ELEV + STRMDIST + RAILROAD + OWLFREQ + $SMCON_{200}$) *p*(PRECIP + DATE + LOWFREQNOISE)	11	7309.51	0.00	0.62
ψ($BRDLF_{400}$ + ELEV + STRMDIST + RAILROAD + $SMCON_{200}$) *p*(PRECIP + DATE + LOWFREQNOISE)	10	7314.02	2.34	0.19
ψ($BRDLF_{400}$ + ELEV + STRMDIST + RAILROAD + OWLFREQ) *p*(PRECIP + DATE + LOWFREQNOISE)	10	7315.68	4.00	0.08
ψ($BRDLF_{400}$ + ELEV + STRMDIST + RAILROAD) *p*(PRECIP + DATE + LOWFREQNOISE)	9	7319.26	5.43	0.04
ψ($BRDLF_{400}$ + ELEV + STRMDIST + OWLFREQ) *p*(PRECIP + DATE + LOWFREQNOISE)	9	7321.08	7.24	0.02
ψ($BRDLF_{400}$ + ELEV + STRMDIST + RAILROAD + OWLFREQ + $SMCON_{200}$) *p*(DATE + LOWFREQNOISE)	10	7319.76	8.08	0.01
ψ($BRDLF_{400}$ + ELEV + RAILROAD + OWLFREQ) *p*(PRECIP + DATE + LOWFREQNOISE)	9	7322.24	8.40	0.01
ψ($BRDLF_{400}$ + ELEV + RAILROAD + OWLFREQ) *p*(PRECIP + DATE + LOWFREQNOISE)	9	7322.24	8.40	0.01

$\psi(BRDLF_{400}$ + $SMCON_{200}$ + RAILROAD + BESTHZ) *p*(PRECIP + DATE + LOWFREQNOISE)	9	7322.47	8.63	0.01
$\psi(BRDLF_{400}$ + $SMCON_{200}$ + STRMDIST + RAILROAD) *p*(PRECIP + DATE + LOWFREQNOISE)	9	7323.37	9.54	0.01
$\psi(BRDLF_{400}$ + ELEV + STRMDIST + RAILROAD + $SMCON_{200}$) *p*(DATE + LOWFREQNOISE)	9	7324.24	10.40	0.00
$\psi(BRDLF_{400}$ + ELEV + STRMDIST + RAILROAD + OWLFREQ) *p*(DATE + LOWFREQNOISE)	9	7325.90	12.07	0.00
$\psi(BRDLF_{400}$ + ELEV + STRMDIST + RAILROAD) *p*(DATE + LOWFREQNOISE)	8	7329.46	13.48	0.00
$\psi(BRDLF_{400}$ + ELEV + STRMDIST + OWLFREQ) *p*(DATE + LOWFREQNOISE)	8	7331.33	15.36	0.00
$\psi(BRDLF_{400}$ + ELEV + RAILROAD + OWLFREQ) *p*(DATE + LOWFREQNOISE)	8	7332.50	16.53	0.00
$\psi(BRDLF_{400}$ + ELEV + RAILROAD + OWLFREQ) *p*(DATE + LOWFREQNOISE)	8	7332.50	16.53	0.00
$\psi(BRDLF_{400}$ + $SMCON_{200}$ + RAILROAD + BESTHZ) *p*(DATE + LOWFREQNOISE)	8	7332.75	16.77	0.00
$\psi(BRDLF_{400}$ + $SMCON_{200}$ + STRMDIST + RAILROAD) *p*(DATE + LOWFREQNOISE)	8	7333.62	17.65	0.00
ψ(1)*p*(PRECIP + DATE + LOWFREQNOISE)	5	7355.18	32.90	0.00
ψ(1)*p*(DATE + LOWFREQNOISE)	4	7365.49	41.12	0.00

[a] The AICc of the top ranked model was 7332.512.

Table S4. Variable description and sample summaries of covariates used in occupancy models for northern saw-whet owl site use (ψ) and detection likelihood (*p*) from passive acoustic monitoring data in southwestern Oregon. Variables were scaled to have a mean of zero and standard deviation (sd) of one unless otherwise noted.

Variable	Description	Parameter	Sample mean ± sd
LOWFREQNOISE	Mean nightly (*p*) or seasonal (ψ) sound levels reflected in dBFS (decibels relative to full scale) across frequency bands 250-1000 Hz. Range: -116.32 to -75.35 dBFS.	*p*, ψ	-108 ± 4.72
PRECIP	Daily precipitation [1] (mm)	*p*	0.93 ± 2.66
TEMP	Daily mean temperature [1] (Celsius)	*p*	13.66 ± 6.14
DATE	Julian day of survey occasion	*p*	132 ± 32 (12 May 2021 ± 32)
MJRHWY	Distance to main highways from ARU station (m)	ψ	12,255.66 ± 7,463.46
UNPAVED	Distance to unpaved roads from ARU station (m)	ψ	216.12 ± 172.09
SCNDHWY	Distance to paved roads from ARU station (m)	ψ	3,118.43 ± 2056.33

RAILROAD	Distance to railroads from ARU station (km)	ψ	16.35 ± 11.55
STRMDIST	Distance to streams from ARU station (m)	ψ	656.16 ± 371.74
RUGGED	Terrain ruggedness calculated as standard deviation of elevation within buffer around ARU station (200, 400, 600 m)	ψ	200: 29.5 ± 9.09<br>400: 48.25± 14.42<br>600: 61.59 ± 17.99
TOPO	Topographic position index (TPI) of station in buffer around ARU station (200, 400, 600 m)	ψ	200: -0.13 ± 0.19<br>400: 0.18 ± 0.21<br>600: 0.12 ± 0.24
ELEV	Mean elevation of the station (m)	ψ	687.64 ± 198.63
SNAG	Mean density of snags (trees per hectare) in buffer around ARU station [2] (200, 400, 600 m)	ψ	200: 17.85 ± 10.88<br>400: 17.27 ± 8.88<br>600: 17.03 ± 8.02
CANCOV	Mean canopy cover of all live trees in buffer around ARU station [2] (200, 400, 600 m)	ψ	200: 71.27 ± 11.27<br>400: 69.43 ± 11.75<br>600: 68.37 ± 11.99
STNDHGHT	Stand height, computed as an average of heights of all dominant and codominant trees in buffer around ARU station [2] (200, 400, 600 m)	ψ	200: 23.43 ± 5.93<br>400: 22.90 ± 5.32<br>600: 22.58 ± 5.09

OGSI80	Old-growth structure index binned for mature forest in our region (≥80yrs). Calculated from abundance of large live trees, snags and down wood, and diversity of tree sizes. Proportion of OGSI80 in buffer around ARU station [3] (200, 400, 600 m)	ψ	200: 0.53 ± 0.29<br>400: 0.50 ± 0.22<br>600: 0.48 ± 0.19
SMCON	Proportion of small conifers in buffer around ARU station (200, 400, 600 m) [2]	ψ	200: 0.35 ± 0.20<br>400: 0.34 ± 0.16<br>600: 0.33 ± 0.14
BRDLF	Proportion of broadleaf trees in buffer around ARU station (200, 400, 600 m) [2]	ψ	200: 0.04 ± 0.08<br>400: 0.05 ± 0.07<br>600: 0.05 ± 0.06
OWLFREQ	Mean nightly sound levels within the saw-whet owl's greatest sensitivity between 1.6-7.1 kHz (dBFS). Range: -117.47 to -106.01 dBFS.	ψ	-112.63 ± 2.25
BESTHZ	Mean nightly sound levels within the saw-whet owl's peak hearing sensitivity at 4kHz (dBFS)	ψ	-112.63 ± 2.36

OWL_LOUD	Proportion of days over -113.27 dBFS (0.50 quantile) calculated between 1.70-7.10 kHz to reflect saw-whet hearing sensitivity.	ψ	0.096 ± 0.11

Table S5. Spearman's correlation table for site covariates used in occupancy models for northern saw whet owls in southwestern Oregon, USA.

n	*Variable*	*1*	*2*	*3*	*4*	*5*	*6*	*7*	*8*	*9*	*10*	*11*	*12*	*13*	*14*	*15*	*16*	*17*	*18*	*19*	*20*	*21*
1	$CANCOV_{400}$	1.00																				
2	$CANCOV_{600}$	0.98	1.00																			
3	$SNAG_{600}$	-0.52	-0.50	1.00																		
4	ELEV	0.27	0.29	0.11	1.00																	
5	UNPAVEDRD	-0.01	-0.01	0.06	-0.07	1.00																
6	RAILROAD	0.11	0.10	-0.07	0.23	-0.13	1.00															
7	$RUGGED_{600}$	0.01	0.00	0.18	0.16	0.15	-0.25	1.00														
8	$RUGGED_{200}$	-0.03	-0.03	0.13	0.04	0.20	-0.17	0.67	1.00													
9	$RUGGED_{400}$	-0.01	-0.02	0.16	0.10	0.19	-0.22	0.90	0.86	1.00												
10	$BRDLF_{200}$	-0.40	-0.40	0.13	-0.14	-0.01	-0.12	0.10	0.12	0.09	1.00											
11	$SMCON_{200}$	0.21	0.21	-0.11	0.04	-0.13	0.15	-0.19	-0.20	-0.19	-0.33	1.00										
12	$BRDLF_{400}$	-0.47	-0.49	0.09	-0.23	0.02	-0.14	0.10	0.15	0.10	0.87	-0.35	1.00									
13	$SMCON_{400}$	0.35	0.35	-0.11	0.11	-0.15	0.26	-0.21	-0.19	-0.20	-0.38	0.86	-0.42	1.00								
14	$BRDLF_{600}$	-0.51	-0.54	0.10	-0.25	0.03	-0.19	0.10	0.14	0.10	0.74	-0.32	0.93	-0.42	1.00							
15	$SMCON_{600}$	0.42	0.44	-0.13	0.13	-0.14	0.33	-0.23	-0.21	-0.22	-0.39	0.71	-0.47	0.93	-0.51	1.00						
16	$TOPO_{200}$	0.02	0.02	0.13	0.20	0.14	0.04	0.11	0.05	0.07	-0.01	-0.52	-0.04	-0.62	-0.06	-0.61	1.00					
17	OWLFREQ	-0.15	-0.16	0.13	0.05	0.18	0.04	0.06	0.13	0.07	0.11	0.03	0.10	0.01	0.08	0.00	-0.09	1.00				
18	BESTHZ	-0.03	-0.04	0.06	0.11	0.11	0.09	0.01	0.01	-0.01	0.07	0.03	0.02	0.02	0.00	0.02	-0.08	0.88	1.00			
19	LOWFREQNOISE	-0.15	-0.16	0.06	0.05	0.28	-0.11	0.22	0.21	0.22	0.15	-0.10	0.22	-0.11	0.19	-0.12	-0.04	0.48	0.23	1.00		
20	OWL_LOUD	-0.04	-0.04	0.13	0.16	0.08	0.06	0.01	0.07	0.02	0.01	0.09	-0.02	0.10	-0.03	0.11	-0.08	0.80	0.70	0.37	1.00	
21	STRMDIST	-0.07	-0.07	0.10	0.17	-0.08	-0.02	0.10	-0.08	0.01	0.04	-0.07	0.06	-0.09	0.08	-0.08	0.05	-0.17	-0.13	0.04	-0.10	1.00

Table S6. Spearman's correlation table for survey covariates used in detection probability models for northern saw-whet owls in southwestern Oregon, USA.

n	*Variable*	*1*	*2*	*3*	*4*
1	LOWFREQNOISE	1.00			
2	PRECIP	0.12	1.00		
3	TEMP	-0.21	-0.47	1.00	
4	DATE	-0.24	-0.13	0.68	1.00

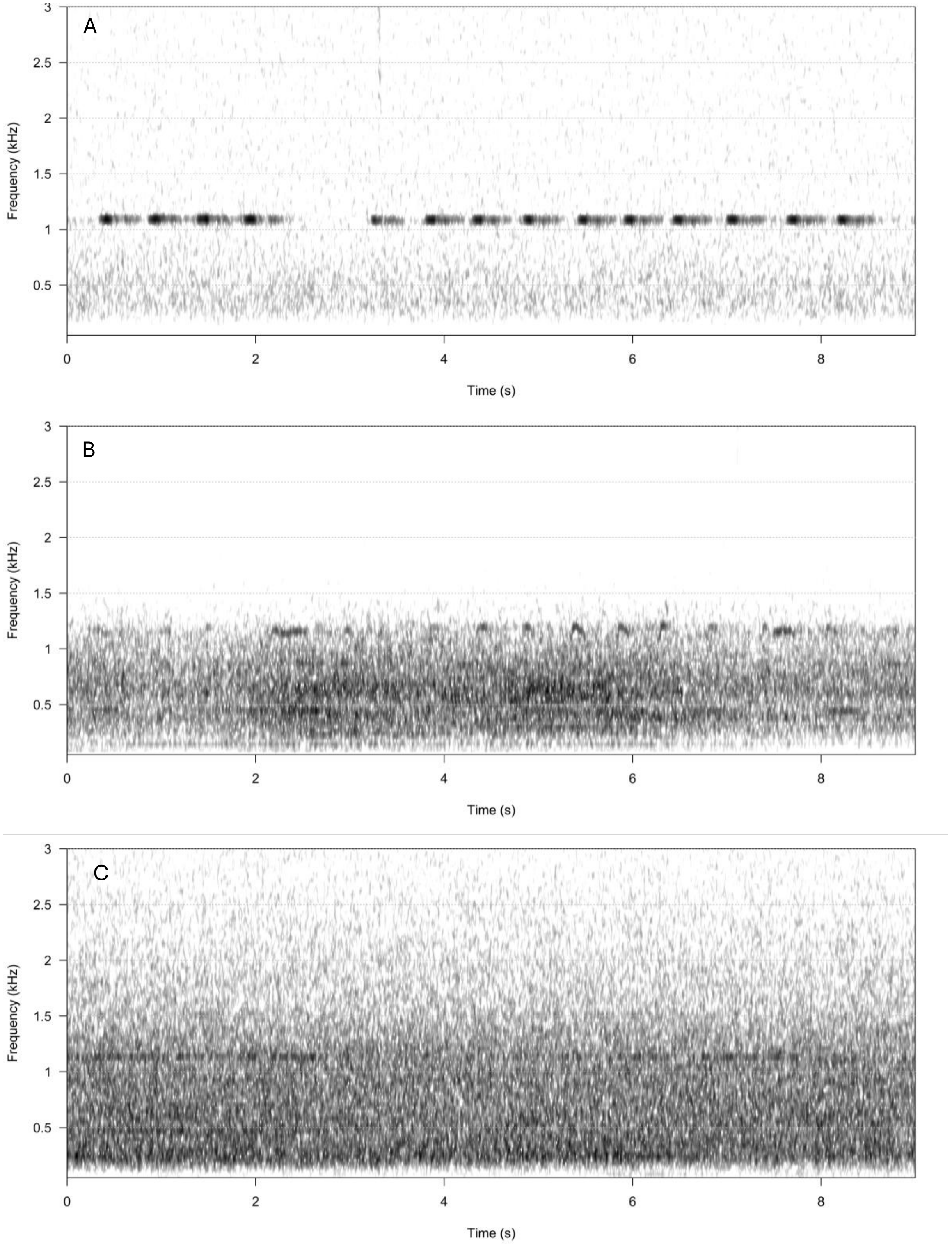

Figure S1. Spectrograms displaying Northern saw-whet owl “toot” vocalization with very little background noise (A) masking low frequency noise cover (B) and masking low-to-mid frequency noise (C).

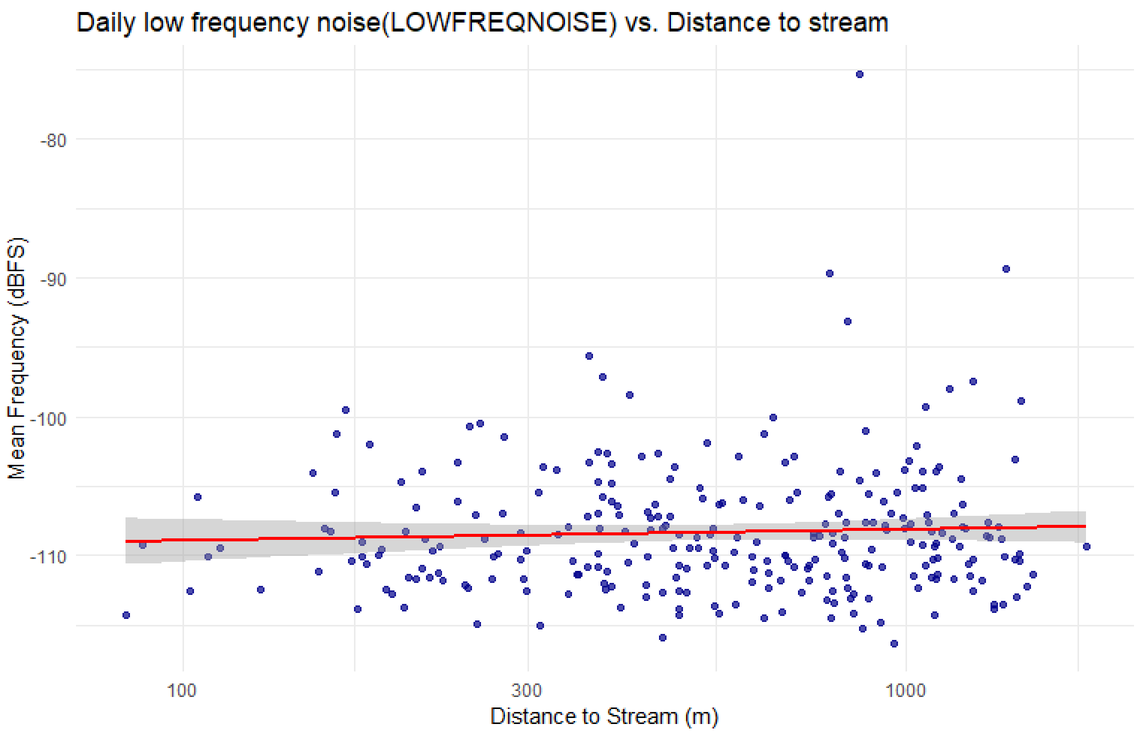

Figure S2. A linear normal model indicated mean low frequency noise (250-1000 Hz) was not significantly related to distance from streams (β = 0.80 ± 0.99 SE, $t = 0.80$, $p = 0.42$, $R^2 = 0.00$).

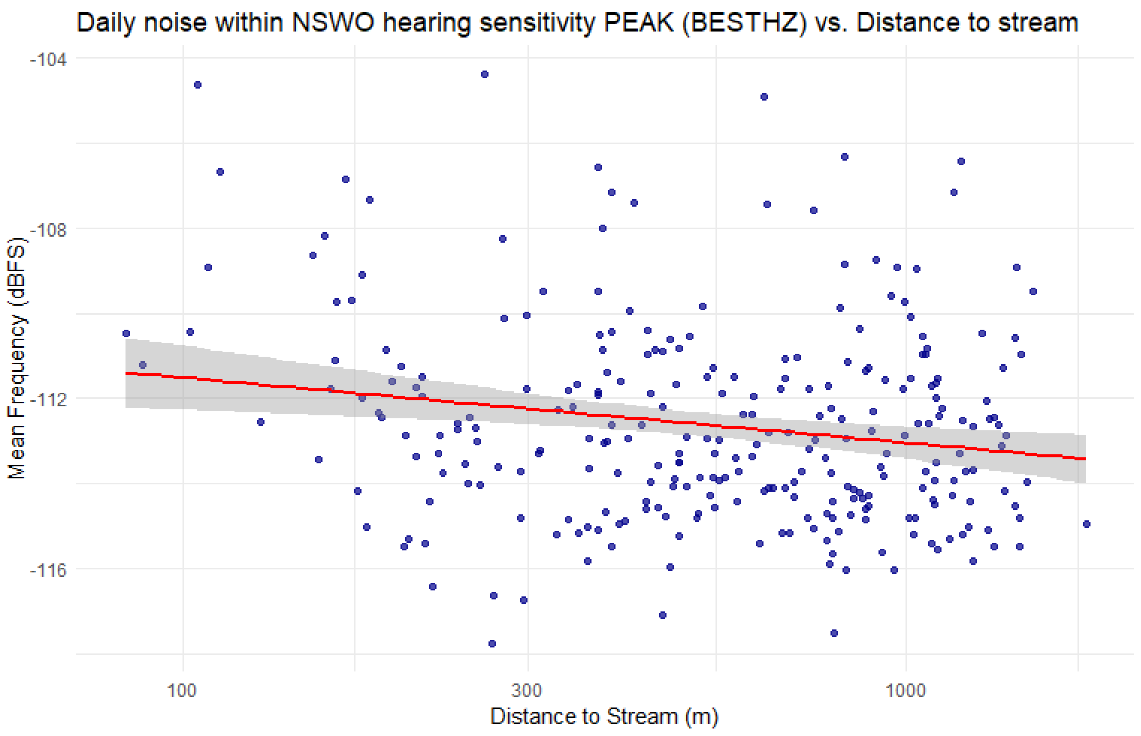

Figure S3. A linear normal model demonstrated mean ambient noise levels at the peak (4kHz) of the Northern saw-whet owl hearing range decreased significantly with increasing distance from streams ($\beta = -1.81 \pm 0.46$ SE, $t = -3.92$, $p < 0.01$, $R^2 = 0.03$)

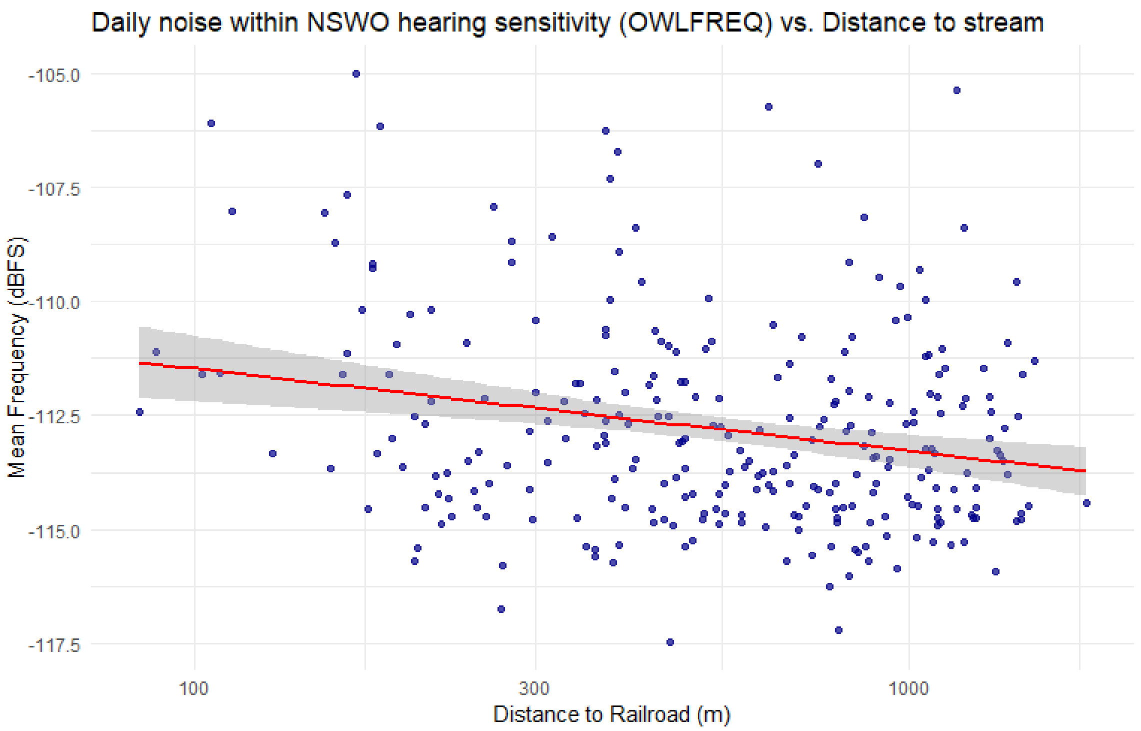

Figure S4. A linear normal model demonstrated mean ambient noise in the northern saw-whet owl hearing range decreased significantly with increasing distance from streams ($\beta = -1.81 \pm 0.46$ SE, $t = -3.92$, $p < 0.001$, $R^2 = 0.05$)

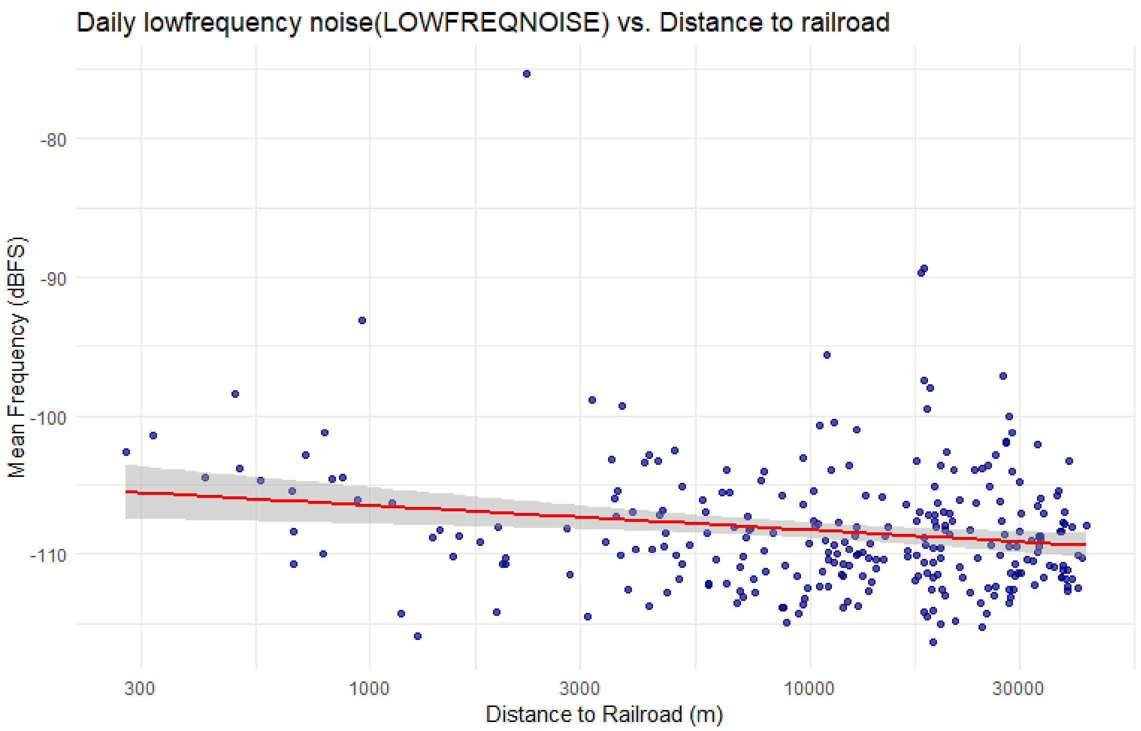

Figure S5. A linear normal model demonstrates that mean low-frequency noise decreased significantly with increasing distance from railroads (β = –1.77 ± 0.59 SE, $t = -3.00$, $p = 0.003$, $R^2 = 0.03$)

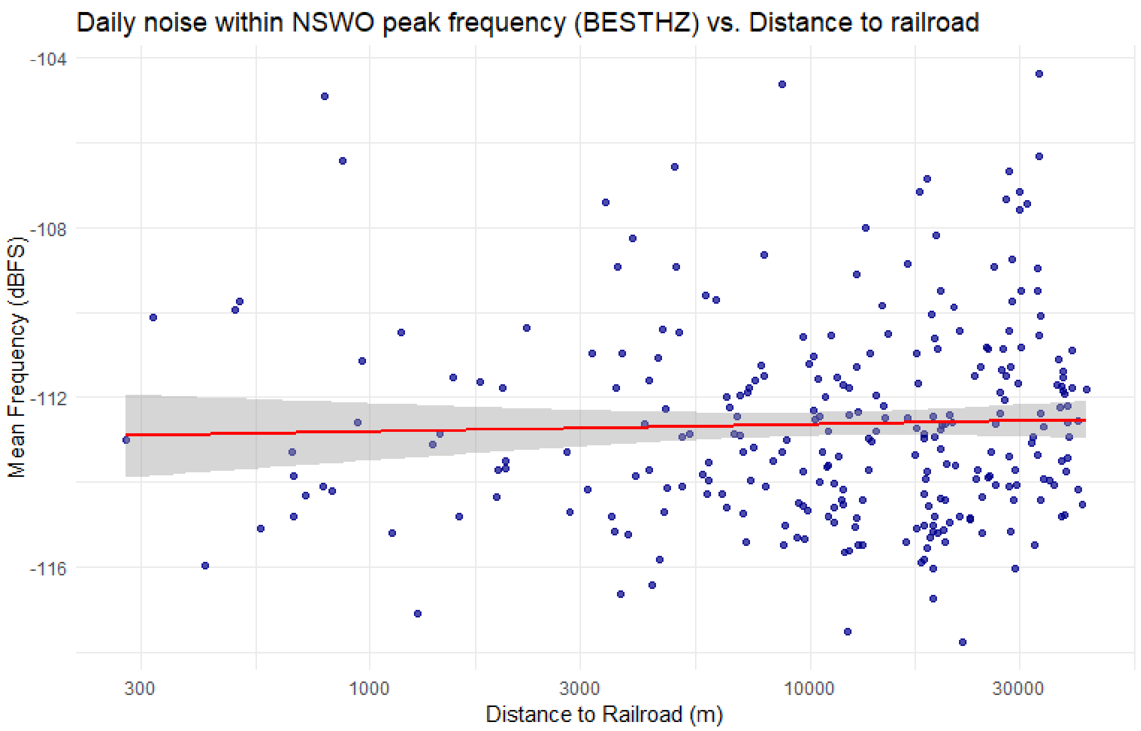

Figure S6. A linear normal model demonstrated mean noise at the peak hearing frequency 4kHz is not significantly related to distance to railroads (β = 0.18 ± 0.30 SE, t = 0.59, p = 0.56, R^2 = 0.00).

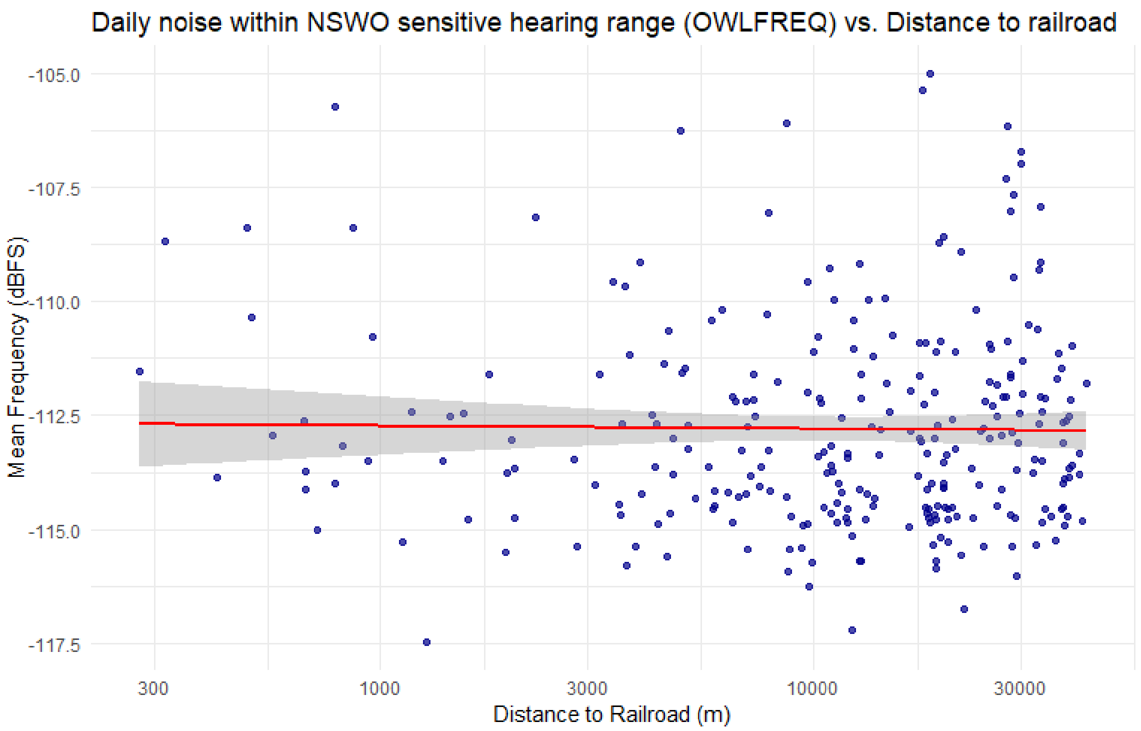

Figure S7. A linear normal model demonstrated mean noise within the most sensitive range of the NSWO's hearing range is not significantly related to distance to railroads (β = –0.07 ± 0.29 SE, t = –0.24, p = 0.81, R^2 = 0.00).

Resources:

1. PRISM Climate Group. Parameter-elevation Regions on Independent Slops Models, Gridded Climate Data. https://prism.oregonstate.edu/.

2. LEMMA Team. Landscape Ecology Modeling, Mapping, and Analysis (LEMMA). *Gradient Nearest Neighbor (GNN) raster dataset (version 2020.01) Modeled forest vegetation data using direct gradient analysis and nearest neighbor imputation.* https://lemma.forestry.oregonstate.edu/data/structure-maps (2020).

3. Davis, R. J. *et al. Northwest Forest Plan–the First 20 Years (1994-2013): Status and Trends of Late-Successional and Old-Growth Forests*. PNW-GTR-911 https://www.fs.usda.gov/treesearch/pubs/50060 (2015) doi:10.2737/PNW-GTR-911.
